# Deficiency of the Mitochondrial Electron Transport Chain in Muscle Does Not Cause Insulin Resistance

**DOI:** 10.1371/journal.pone.0019739

**Published:** 2011-05-12

**Authors:** Dong-Ho Han, Chad R. Hancock, Su Ryun Jung, Kazuhiko Higashida, Sang Hyun Kim, John O. Holloszy

**Affiliations:** Division of Geriatrics and Nutritional Sciences, Department of Medicine, Washington University School of Medicine, Saint Louis, Missouri, United States of America; New Mexico State University, United States of America

## Abstract

**Background:**

It has been proposed that muscle insulin resistance in type 2 diabetes is due to a selective decrease in the components of the mitochondrial electron transport chain and results from accumulation of toxic products of incomplete fat oxidation. The purpose of the present study was to test this hypothesis.

**Methodology/Principal Findings:**

Rats were made severely iron deficient, by means of an iron-deficient diet. Iron deficiency results in decreases of the iron containing mitochondrial respiratory chain proteins without affecting the enzymes of the fatty acid oxidation pathway. Insulin resistance was induced by feeding iron-deficient and control rats a high fat diet. Skeletal muscle insulin resistance was evaluated by measuring glucose transport activity in soleus muscle strips. Mitochondrial proteins were measured by Western blot. Iron deficiency resulted in a decrease in expression of iron containing proteins of the mitochondrial respiratory chain in muscle. Citrate synthase, a non-iron containing citrate cycle enzyme, and long chain acyl-CoA dehydrogenase (LCAD), used as a marker for the fatty acid oxidation pathway, were unaffected by the iron deficiency. Oleate oxidation by muscle homogenates was increased by high fat feeding and decreased by iron deficiency despite high fat feeding. The high fat diet caused severe insulin resistance of muscle glucose transport. Iron deficiency completely protected against the high fat diet-induced muscle insulin resistance.

**Conclusions/Significance:**

The results of the study argue against the hypothesis that a deficiency of the electron transport chain (ETC), and imbalance between the ETC and β-oxidation pathways, causes muscle insulin resistance.

## Introduction

There has been much interest in the possibility that the insulin resistance in obese individuals and patients with type 2 diabetes is mediated by mitochondrial deficiency in skeletal muscle [1]–[10]. This possibility was suggested by the finding that obese insulin resistant individuals and type 2 diabetics generally have about 30% less mitochondria in their skeletal muscles than age-matched individuals with normal insulin action [1]–[7]. The mechanism by which “mitochondrial deficiency” is thought to cause insulin resistance is accumulation of intramuscular lipids as a result of a decreased capacity for fat oxidation [2], [9]. There are a number of reasons why this hypothesis is unlikely to be correct, including the fact that diabetic muscle can increase fat oxidation many-fold in response to exercise, indicating that a 30% decrease in mitochondria is not limiting for fat oxidation at rest [11]. However, Ritov et al. [12] have proposed the new concept that insulin resistance is not due to a decrease in skeletal muscle mitochondria per se but to a selective decrease in the components of the mitochondrial electron transport chain, with no decrease in the enzymes of the mitochondrial fatty acid oxidation pathway. They proposed that, because of this discrepancy, toxic products of incomplete fatty acid oxidation accumulate and mediate skeletal muscle insulin resistance [12].

The purpose of the present study was to test this hypothesis. For the experimental model, we used rats made severely iron deficient. Iron deficiency results in a decrease in the iron-containing constituents of the mitochondrial respiratory chain without affecting the enzymes of the fatty acid oxidation pathway [13]. As a model of skeletal muscle insulin resistance we used rats fed a high fat diet, which rapidly develop what appears to be the rodent equivalent of the visceral obesity/metabolic/insulin-resistance syndrome [14]–[17].

Iron deficiency could have effects on the liver, pancreatic beta cells, adipose tissue and brain that might affect insulin production, whole body insulin action and glucose uptake and metabolism. Such effects may be interesting, but have no direct relevance to the hypothesis being evaluated in this study, which involves only skeletal muscle. This study, therefore, deals only with the effect of an imbalance between the mitochondrial fatty acid oxidation pathway and the capacity of the electron transport chain on insulin-stimulated glucose transport in skeletal muscle.

## Results

### Body weights

The high fat diets resulted in a significant increase in weight gain (Table 1). Iron deficiency partly protected against the increase in plasma insulin induced by feeding a high fat diet.

**Table 1:** Body weights and glucose and insulin levels.

	Diets			
	Low Fat Normal Iron	Low Fat Iron Deficient	High Fat Normal Iron	High Fat Iron Deficient
Body Weights, g n = 11	440±5	426±9	477±11*	465±16*
Glucose mg/dl n = 10	120±3	132±3	139±7	140±13
Insulin, µU/ml n = 6	21.3±0.8	---	69±6.2†	34.4±2.5

Values are means ± SE. n  =  number of rats per group.

*High Fat versus Low Fat Diet, P<0.01.

† High Fat Normal Iron versus Low Fat Normal Iron and High Fat Iron Deficient, P<0.01.

### Muscle triglycerides

**Figure 1:**
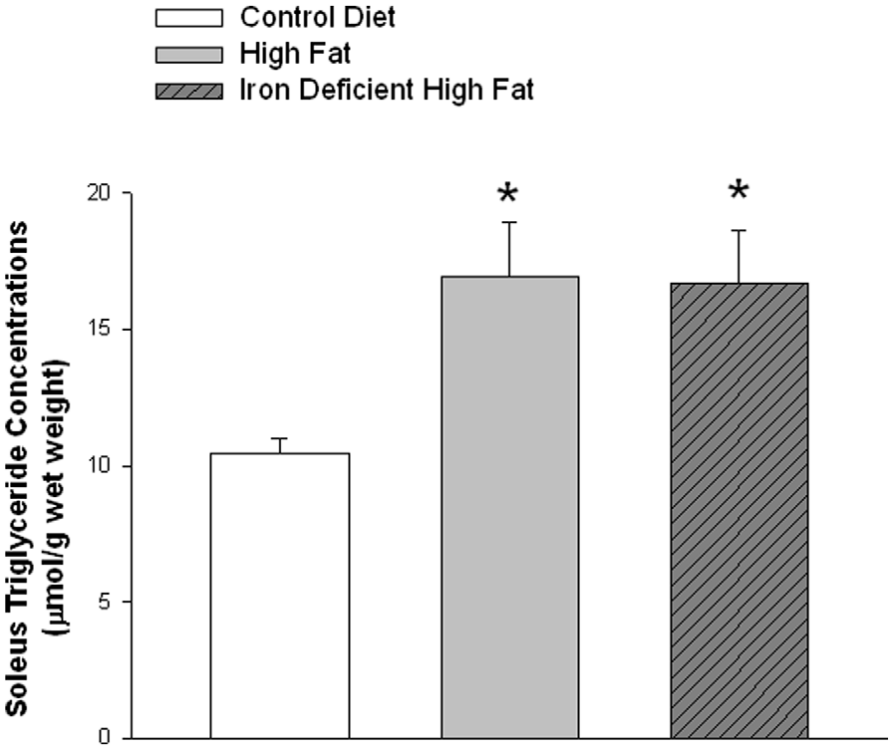
Triglyceride concentration in soleus muscles. Values are means ± SE for 8 muscles per group. High fat diet groups versus control diet group, P<0.01.

As in previous studies [18], [19], the high fat diet resulted in significant increases in muscle triglyceride concentration in the two high fat diet groups (Fig. 1).

### Muscle glucose transport

**Figure 2:**
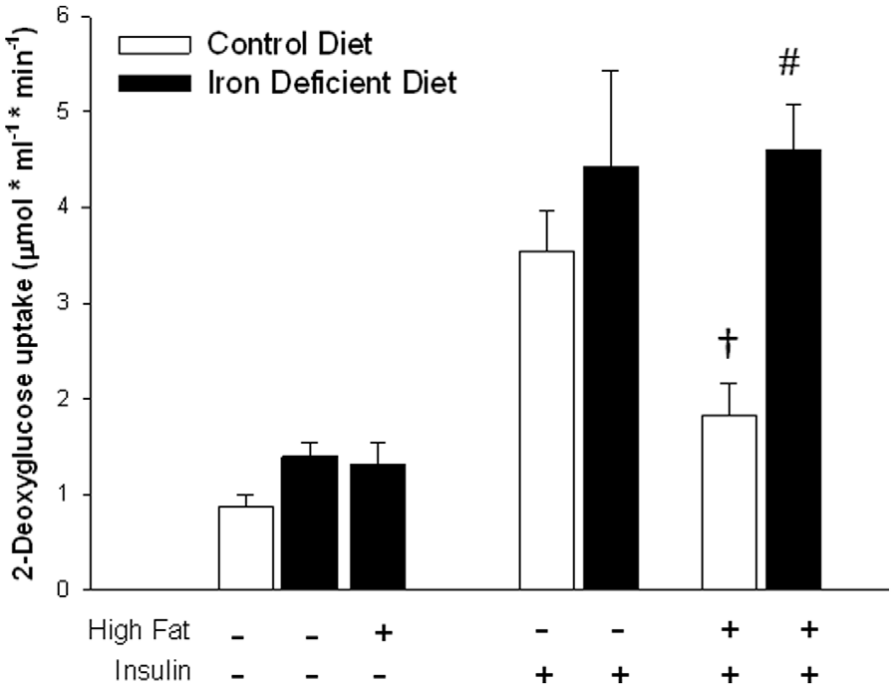
Maximally insulin stimulated (2 mU insulin/ml) glucose transport activity. 2-Deoxy-[^3^H] glucose uptake was measured in soleus muscle strips in vitro. Values are means ± SE for 8 muscles per group except for the low fat iron deficient diet groups, in which there were 4 muscles per group. ^†^High Fat diet, Normal Iron group versus Low Fat diet, Normal Iron group, P<0.01. ^#^Insulin stimulated Iron-Deficient, High Fat diet group versus Insulin-Stimulated Normal Iron, Low Fat diet group, P<0.05.

The high fat diet resulted in severe insulin resistance of muscle glucose transport. As shown in Fig. 2, iron deficiency not only completely protected against the high fat diet-induced muscle insulin resistance, but also resulted in somewhat higher glucose transport rates compared to muscles from rats on the normal iron-containing diets.

### Fatty acid oxidation by muscle

**Figure 3:**
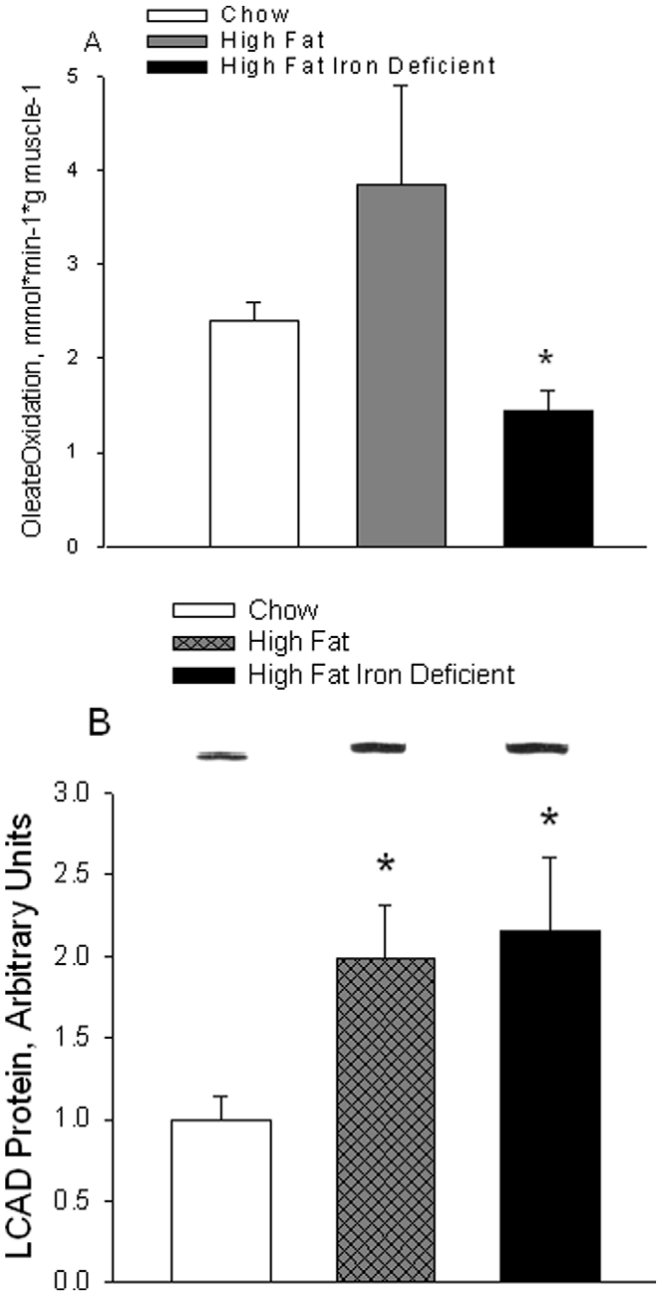
Oleate oxidation by triceps muscle homogenates. A. The capacity to oxidize fatty acids was evaluated by measuring the rate of CO_2_ production from [^14^C] oleate under conditions in which ADP and Pi are not limiting. Bars are means ± SE for 7 to 8 muscles per group. *High Fat, Iron-Deficient diet versus other groups, P<0.05. B. Long chain acyl-CoA dehydrogenase (LCAD) protein expression in triceps muscle was measured by Western blot analysis. *High Fat diets versus control Low Fat diet, P<0.01.

The capacity to oxidize fatty acids was evaluated by measuring the rate of ^14^CO_2_ production from [^14^C] oleate by whole homogenates of triceps muscle under conditions in which ADP and Pi are not rate-limiting. In previous studies, it was found that high fat diets induce an increase in the ability of skeletal muscle to oxidize fatty acids [20]–[22], In the present study, the rate of oleate oxidation by triceps muscles was 60% higher in the high fat, normal iron, than in the low fat, normal iron control group (Fig. 3A). However, this difference did not attain statistical significance (P = 0.08) because of a large standard deviation. Oleate oxidation was markedly reduced in muscle of the high fat diet, iron deficient group (Fig. 3A). As shown in Fig. 3B, long chain fatty acyl-CoA dehydrogenase (LCAD) protein expression, which was used as a marker for the capacity of the β-oxidation pathway, was increased about 2-fold in muscles from both the normal iron and the iron-deficient high fat diet fed rats. Expression of the enzymes of the fatty acid oxidation pathway is coordinately regulated by PPARδ and PPARα, transcription factors that are activated by fatty acids, so LCAD can be used as a marker for the capacity of the mitochondrial fatty acid oxidative enzyme pathway [23].

### PGC-1α response

**Figure 4:**
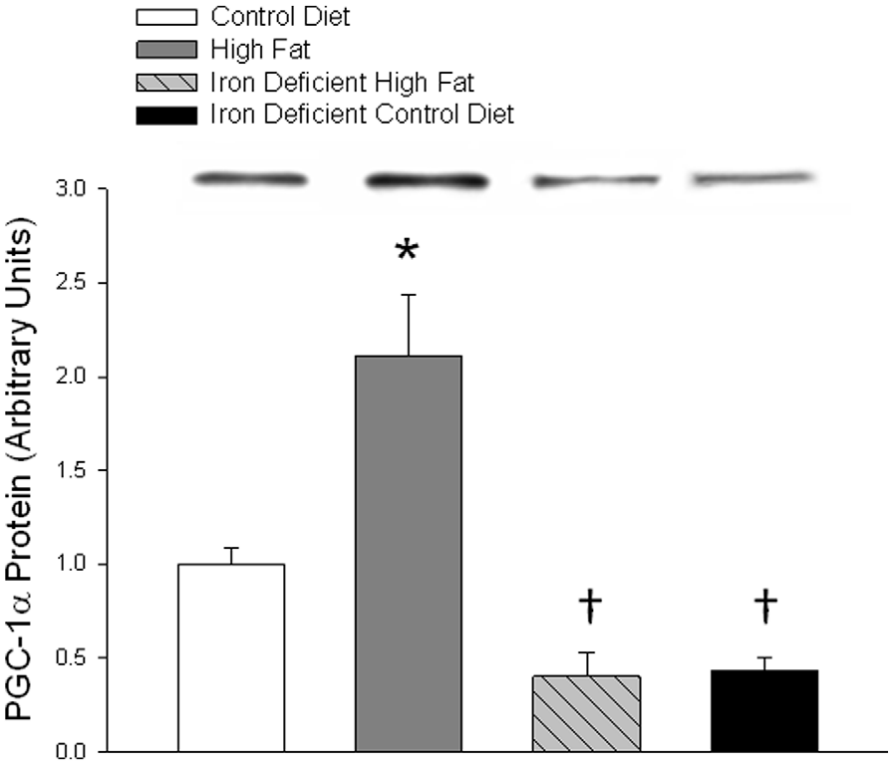
PGC-1α expression in soleus muscle was measured by Western blot analysis. Bars are means ± SE for 5 to 7 muscles per group. *High Fat diet versus Low Fat control diet group, P<0.01. ^†^Iron deficient diet groups versus normal iron groups, P<0.01.

Feeding rats a high fat diet induces an increase in PGC-1α protein expression in muscle [21], [22], [24]. This increase in PGC-1α occurs gradually over about a 4 wk period and is mediated by a post transcriptional effect that appears to result from PPARδ activation [22]. As shown in Fig. 4, five weeks of high fat diet feeding resulted in a significant increase in PGC-1α protein expression in soleus muscle. The iron deficient diets resulted in an unexpected, large decrease in PGC-1α protein. This finding came as a surprise, as there is no obvious reason why iron deficiency should affect PGC-1α protein level.

### Mitochondrial proteins and GLUT4

It was previously found that severe iron deficiency, similar to that induced in the present study, has no effect on the levels of enzymes of the mitochondrial fatty acid oxidation pathway or on non-iron containing enzymes of the citrate cycle [13]. The fatty acid oxidation and citrate cycle enzymes measured included carnitine palmitoyl transferase, 3-hydroxyacyl-CoA dehydrogenase, 3-ketoacid CoA thiolase, citrate synthase, isocitrate dehydrogenase, and fumarase [13]. There were large decreases in iron-containing compounds of the electron transport chain [13].

**Figure 5:**
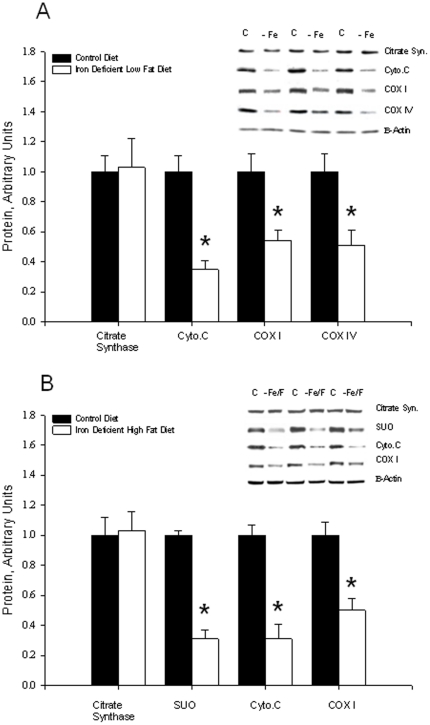
Effects of iron deficiency on expression of citrate synthase and iron-containing mitochondrial respiratory chain proteins in soleus muscles of A) Low Fat and B) High Fat diet groups. Bars are means ± SE for 7 to 8 muscles per group. *Iron Deficient versus Normal Iron, Low Fat control diet group, P<0.01.

In the present study the expression of citrate synthase, a non-iron containing enzyme of the citrate cycle, was unaffected by the iron deficiency in soleus muscle in both low-fat (Fig. 5A) and high fat diet groups (Fig. 5B). As in previous studies [13], [25], iron deficiency resulted in a marked decrease in expression of a number of representative iron-containing proteins of the mitochondrial respiratory chain. This effect was present in both the low fat (Fig. 5A) and high fat (Fig. 5B) diet groups. Expression of the glucose transporter GLUT4 in skeletal muscle, was unaffected by iron deficiency (Fig. 5A and B). Similar effects of iron deficiency were observed in triceps and gastrocnemius muscles (data not shown).

### Ratios of LCAD to electron transport chain markers

**Figure 6:**
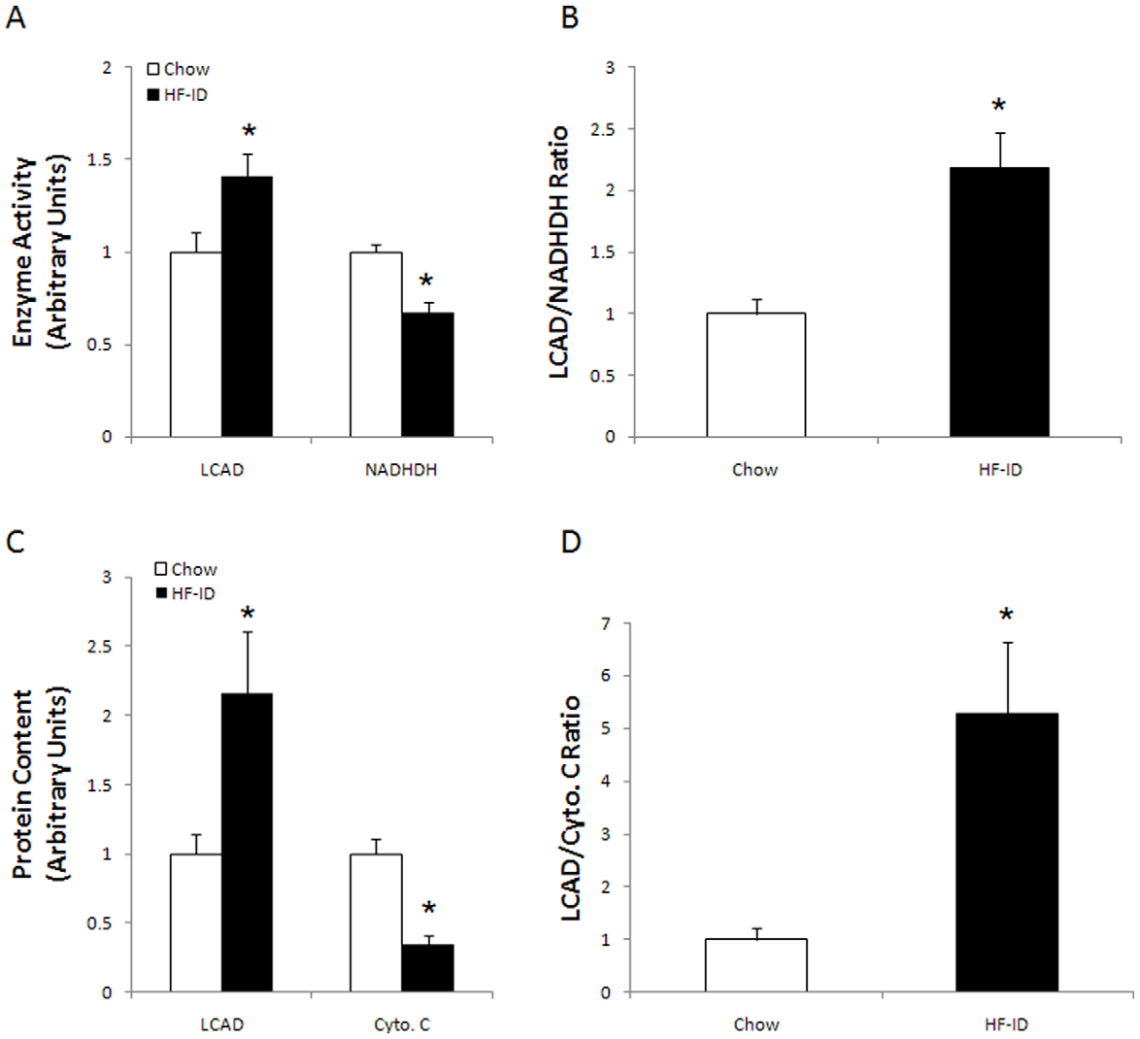
Ratios of long chain acyl CoA dehydrogenase (LCAD) to NADH dehydrogenase (NADHDH) in soleus muscle. A. LCAD and NADHDH enzyme activity levels. B. Ratio of LCAD activity to NADHD activity. C. LCAD and cytochrome c (Cyto c) protein levels. D. Ratios of LCAD to Cyto c protein levels, *P<0.05.

To evaluate the magnitude of the imbalance between the fatty acid oxidation and the ETC created by iron deficiency and a high fat diet, we measured the enzyme activities of LCAD and the iron containing, electron transport chain enzyme NADH dehydrogenase. As shown in Figure 6A, there was an increase in LCAD activity and a large decrease in the activity of NADH dehydrogenase in muscle of the iron deficient, high fat diet fed rats. As a result, there was a more than 2-fold increase in the ratio of LCAD activity, representative of the fatty acid oxidation pathway, to NADH dehydrogenase, representative of the ETC (Fig. 6B). A similar change occurred in the ratio of LCAD protein to cytochrome c protein (Fig. 6D).

### AMP-activated protein kinase (AMPK) phosphorylation

**Figure 7:**
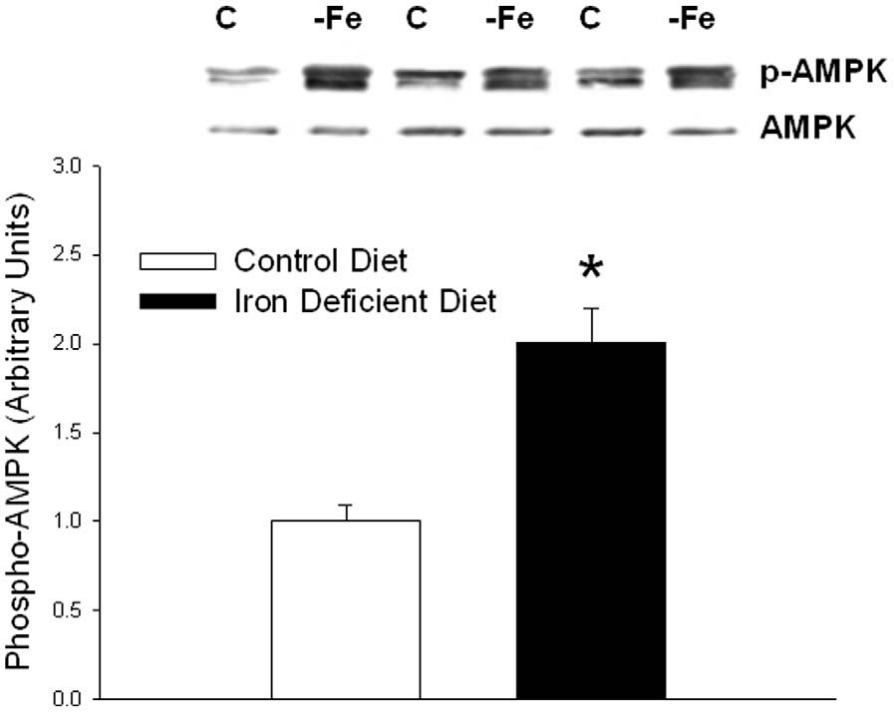
AMP-kinase phosphorylation was measured by Western blot analysis. Each bar represents the mean ± SE for 12 muscles per group. *Iron Deficient versus Normal Iron control diet, P<0.01.

It seemed possible that, as in previous studies in which mitochondrial dysfunction in muscle resulted in an increase in glucose transport activity [26], [27], there might be an increase in AMPK activity in the iron-deficient muscles. This turned out to be the case as evidenced by a significant increase in AMPK phosphorylation in the iron-deficient muscles (Fig. 7). There was no difference in AMPK phosphorylation between the iron-deficient high fat diet and the iron-deficient chow diet groups, so the data on these groups was combined under “iron-deficient diet”.

## Discussion

Patients with type 2 diabetes and obese, insulin resistant individuals generally have 30% less mitochondria in their muscles than normal age-matched individuals [2], [4], [5]. This finding led to the hypothesis that a reduction in mitochondrial content of skeletal muscle impairs the ability of muscle to oxidize fat, resulting in lipid accumulation and insulin resistance [2], [9]. There are a number of arguments against this concept (reviewed in [11]. The rate of substrate utilization is determined by the rate of ATP utilization, which is low in resting muscle. Even patients with type 2 diabetes with a low exercise capacity are able to increase the rate of fat oxidation by muscle many fold in response to exercise. It, therefore, seems improbable that a 30% decrease in muscle mitochondria could limit fat oxidation in resting muscle. Furthermore, a decrease in mitochondria severe enough to limit fat oxidation would result in compensatory increase in glucose uptake and utilization by muscle, not insulin resistance.

The finding that rats fed high fat diets develop insulin resistance despite a concomitant increase in muscle mitochondria and in the ability to oxidize fat [21], [22] also argues against the concept that a decrease in mitochondria mediates insulin resistance. Direct evidence against the mitochondrial dysfunction causes insulin resistance hypothesis is provided by the results of studies in rodents showing that severe impairment of mitochondrial function in muscle results in increased basal and insulin-stimulated muscle glucose uptake [26]–[28]. Furthermore, Asian Indians with severe insulin resistance and diabetes have an enhanced muscle mitochondrial capacity for ATP production [29], and weight loss induced by bilio-pancreatic diversion that results in reversal of insulin resistance and type 2 diabetes is not associated with an increase in muscle mitochondria [30]. The effect of impaired mitochondrial function has also been studied in vitro by Brown et al. [31], who incubated human muscle cell cultures with sodium azide and found that a ∼50% decrease in respiration increased insulin independent glucose transport and had no effect on insulin-stimulated glucose transport or Akt phosphorylation.

However, these arguments do not apply to a new version of the mitochondrial deficiency hypothesis proposed by Ritov et al. [12]. These investigators found that muscle mitochondria of type 2 diabetic patients had a significantly reduced activity of NADH oxidase and electron transport chain (ETC) activity but no deficiency of citrate synthase or β-hydroxyacyl-CoA dehydrogenase activities. Based on these findings, Ritov, et al. [12] hypothesized that “a deficiency of ETC and disbalance between ETC and β-oxidation” causes insulin resistance due to accumulation of toxic byproducts of incomplete oxidation of fat.

The purpose of the present study was to test the hypothesis of Ritov et al. [12] that insulin resistance is caused by a reduced capacity of the mitochondrial electron transport chain relative to the capacity of the fatty acid β-oxidation pathway. In our experimental model, rats that were made severely iron deficient and fed a high fat diet, the imbalance between the capacity for β-oxidation of fatty acids and the capacity of the electron transport chain in muscle was considerably more severe than that reported for human diabetic muscle by Ritov et al. [12]. Not only were the levels of the ETC proteins cytochrome c and succinate ubiquinone oxidoreductase markedly reduced, but LCAD, which we used as a marker for the fatty acid β-oxidation pathway, was increased ∼2-fold in the high fat diet fed rats. Similar to these changes in mitochondrial protein levels, the ratio of LCAD enzyme activity to NADH dehydrogenase activity was ∼2-fold higher in iron-deficient than in control muscles. Expression of the enzymes of the β-oxidation pathway is coordinately regulated by the nuclear receptors PPARδ and α which are activated by fatty acids, and LCAD is, therefore, a marker for the response of the fatty acid (FA) oxidation pathway [23]. Despite this large imbalance between the capacities for fatty acid β-oxidation and the ETC, the iron deficient muscles not only did not become insulin resistant, but were completely protected against the insulin resistance induced by a high fat diet. We think that the present results, together with those of Wrendenberg et al. [27], disprove the concept that a reduction in ETC capacity relative to the capacity of the fatty acid β-oxidation pathway causes insulin resistance. It should also be noted that, in contrast to the report by Ritov et al. [12], a number of other studies have found that the remaining mitochondria in muscles of diabetic and insulin-resistant obese humans have normal function [32], [33], and that mitochondria of insulin resistant [24], [34] and diabetic [34] rodent muscles also have normal function.

As in previous studies of the effects of mitochondrial dysfunction [26], [27], AMP kinase was activated in the iron deficient muscles. AMPK activation results in an increase in glucose transport activity in muscle that is additive to the effect of insulin [35]–[39]. This probably explains why glucose transport was higher in the iron deficient than in the normal iron muscles.

A high fat diet or raising plasma FFA level induces an increase in mitochondrial biogenesis [20]–[22] that is mediated by activation of PPARδ, which brings about a post translational increase in PGC-1α protein [22]. Furthermore, PGC-1α is activated via phosphorylation by AMPK [40], which would normally induce an increase in GLUT4 [41]–[43]. We, therefore, expected that non-iron containing mitochondrial proteins and GLUT4 would be increased in the iron deficient muscles. However, this adaptation was prevented by a large decrease in PGC-1α protein. This finding came as a surprise, as we knew of no evidence that iron plays a role in regulating PGC-1α expression. However, a literature search turned up a paper by Ishii et al. [44] showing that prevention of iron uptake by chelation of iron resulted in inhibition of PGC-1β expression in osteoclasts, while treatment with transferrin increased PGC-1β expression. Our finding of a marked reduction in PGC-1α expression in iron deficient muscles provides evidence that iron availability similarly regulates PGC-1α expression in muscle, and explains why citrate synthase and GLUT4 expression were not increased. It is interesting in the present context that muscle specific PGC-1α knockout mice that were expected to be insulin resistant because of a decrease in respiratory chain proteins, were found to be considerably more insulin sensitive than wild type mice as reflected in glucose uptake during a hyperinsulinemic clamp [45].

In conclusion, the results of this study argue against the hypothesis of Ritov et al. [12] that “a deficiency of electron transport chain (ETC) and disbalance between ETC, β-oxidation and TCA cycle” causes muscle insulin resistance, and provide further evidence that mitochondrial deficiency/dysfunction does not mediate muscle insulin resistance.

## Methods

### Animal Care

This work was approved by the Animal Studies Committee of Washington University School of Medicine (Animal Welfare Assurance #A-3381-01; Approval Number: 20090104). Male Wistar rats weighing ∼50 g were individually housed with a 12∶12 hr light/dark cycle. The animals were given ad libitum access to water and food.

### Diets

The diets were obtained from Harlan Teklad (Madison, WI). The iron deficient diets were made with low-iron casein and iron-deficient mineral mix. The iron deficient low-fat diet contained ∼4.0 kcal/g with 11% of calories from fat. The iron-deficient high fat diet contained ∼5 kcal/g with 50% of calories provided by fat (43% lard, 7% soybean oil). The normal iron high fat diet had the same protein, carbohydrate and fat content as the iron deficient high fat diet.

### Study Design

The rats were assigned to two groups and fed either the iron containing or the iron deficient low fat diets for nine weeks. Fifty percent of the animals on the iron containing diet were then started on the iron containing high fat diet, and fifty percent of the rats that had been on the iron-deficient diet were started on the iron deficient high fat diet. After five weeks on the high fat or low fat diets, the rats were used for measurement of muscle glucose transport and mitochondrial oxidative capacity and respiratory chain protein levels.

### Treatment of animals

After an overnight fast that began at 1800 hr, rats were anesthetized with sodium pentobarbital 50 mg/100 g body weight between 10:00 am and 11:00 am. A week before the rats were killed, blood was obtained from a tail vein between 10:00 am and 11:00 am for measurement of glucose and insulin. The soleus and triceps muscles were dissected out and used for measurement of glucose transport (soleus) and oleate oxidation (triceps), and the gastrocnemius muscle was clamp frozen in situ. Samples for future analysis were stored at −80°C.

### Measurement of glucose transport activity

Soleus muscles were split longitudinally into strips to allow adequate diffusion of oxygen and substrates [46]. One strip was used for measurement of glucose transport activity and the remaining portion of soleus muscle was frozen for later measurement of mitochondrial proteins. The soleus muscle strips were incubated with shaking at 30°C for 1 hr in 2.0 ml of oxygenated Krebs-Henseleit buffer (KHB) supplemented with 8 mM glucose, 32 mM mannitol, and 0.1% bovine serum albumin with or without 2 mU/ml insulin (a maximally effective concentration). Muscles were then washed for 10 min in KHB containing 40 mM mannitol, with or without 2 mU/ml insulin to remove glucose from the extracellular space. Muscles were then incubated for 20 min in 1.0 ml of KHB containing 4 mM 2-deoxy-[^3^H] glucose (2DG) (American Radiolabeled Chemicals, St. Louis, MO) (1.5 µCi/ml) and 36 mM [^14^C] mannitol (ICN Radiochemicals, Irvine, CA) (0.2 µCi/ml) and 2 mU/ml insulin if it was present in the previous incubations, to measure 2-DG transport rates [47]. The gas phase throughout was 95% O_2_-5% CO_2_. Intracellular 2-DG accumulation and extracellular space were determined as described previously [47], [48].

### Measurement of oleate oxidation

Triceps muscles were minced and homogenized in ice-cold 300 mM sucrose containing 10 mM TrisCl and 2 mM EDTA using a Potter-Elveheim glass homogenizer to give a 5% homogenate. Oleate-1-^14^C oxidation by whole homogenates was measured as described previously for [^14^C] palmitate [20].

### Measurement of muscle triglycerides

Muscle triglyceride concentration was determined by extracting total lipids from clamp-frozen soleus muscle samples with chloroform-methanol (2∶1 vol.vol) as described by Folch et al [49], separating the chloroform and methanol-water phases, removing phospho-lipids, and further processing the sample using Frayn and Maycock's [50] modification of the method of Denton and Randle [51]. Triglycerides were then quantified spectrophotometrically as glycerol using an enzymatic assay kit (Sigma Chemical).

### Western blotting

Muscles were homogenized in ice-cold buffer containing 250 mM sucrose, 10 mM HEPES/1 mM EDTA (pH 7.4), 1 mM each of Pefabloc (Roche), EDTA, and NaF, 1 µg/ml of aprotinin, leupeptin, and pepstatin, 0.1 mM bpV(phen), and 2 mg/ml β-glycerophosphate. Homogenates were subjected to three freeze/thaw cycles and centrifuged for 10 min at 700× g. Protein concentration was determined using the Lowry Method. Aliquots were solubilized in Laemmli buffer and subjected to SDS/PAGE. The following antibodies were used for immunoblotting: succinate-ubiquinone oxidoreductase (SUO), cytochrome oxidase subunit 1 (COX1), cytochrome oxidase subunit IV (COXIV) (Molecular Probes), citrate synthase (CS) (Alpha Diagnostic), cytochrome c (cyt c) (Phar-Mingen International), long chain acyl-CoA dehydrogenase (LCAD) (a gift from Dan Kelly), peroxisome proliferator-activated receptor δ coactivator-1α (PGC-1α), adenosine monophosphae kinase AMPK and phospho AMPK (Cell Signaling, Beverly, MA) and GLUT4 (a gift from Mike Mueckler). After incubation with the appropriate secondary antibodies, bands were visualized by ECL and quantified by densitometry.

### Measurement of enzyme activities

Long chain Acyl-CoA dehydrogenase activity was measured as described by Ijlst and Wanders [52]. NADH dehydrogenase activity was measured using an enzyme activity microplate assay kit (Mito Sciences, Eugene, OR).

### Statistical analysis

Results are expressed as means ± SE. The significance of differences between two groups was assessed using Student's t-test. For multiple comparisons, significance was determined by one-way analysis of variance followed by a post hoc comparison using Tukey significant difference method.
